# Inhibition of Interleukin‐6/glycoprotein 130 signalling by Bazedoxifene ameliorates cardiac remodelling in pressure overload mice

**DOI:** 10.1111/jcmm.15147

**Published:** 2020-03-12

**Authors:** Wei Shi, Haiyan Ma, Tianshu Liu, Dan Yan, Pengcheng Luo, Maocai Zhai, Jingwen Tao, Shengqi Huo, Junyi Guo, Chenglong Li, Jiayuh Lin, Cuntai Zhang, Sheng Li, Jiagao Lv, Li Lin

**Affiliations:** ^1^ Division of Cardiology Department of Internal Medicine Tongji Hospital Tongji Medical College Huazhong University of Science and Technology Wuhan China; ^2^ Division of Cardiology Department of Internal Medicine First People's Hospital of Shangqiu Shangqiu China; ^3^ Department of Geriatrics Tongji Hospital Tongji Medical College Huazhong University of Science and Technology Wuhan China; ^4^ Department of Medicinal Chemistry College of Pharmacy University of Florida Gainesville FL USA; ^5^ Department of Biochemistry and Molecular Biology University of Maryland School of Medicine Baltimore MD USA

**Keywords:** Bazedoxifene, cardiac remodelling, interleukin‐6, transverse aortic constriction

## Abstract

The role of IL‐6 signalling in hypertensive heart disease and its sequelae is controversial. Our group demonstrated that Bazedoxifene suppressed IL‐6/gp130 signalling in cancer cells but its effect on myocardial pathology induced by pressure overload is still unknown. We explored whether Bazedoxifene could confer benefits in wild‐type C57BL/6J mice suffering from transverse aortic constriction (TAC) and the potential mechanisms in H9c2 myoblasts. Mice were randomized into three groups (Sham, TAC, TAC+Bazedoxifene, n = 10). Morphological and histological observations suggested TAC aggravated myocardial remodelling while long‐term intake of Bazedoxifene (5 mg/kg, intragastric) attenuated pressure overload‐induced pathology. Echocardiographic results indicated Bazedoxifene rescued cardiac function in part. We found Bazedoxifene decreased the mRNA expression of IL‐6, MMP2, Col1A1, Col3A1 and periostin in murine hearts after 8‐week surgery. By Western blot detection, we found Bazedoxifene exhibited an inhibition of STAT3 activation in mice three hours and 8 weeks after TAC. Acute TAC stress (3 hours) led to down‐regulated ratio of LC3‐Ⅱ/LC3‐Ⅰ, while in mice after long‐term (8 weeks) TAC this ratio becomes higher than that in Sham mice. Bazedoxifene inverted the autophagic alteration induced by TAC at both two time‐points. In H9c2 myoblasts, Bazedoxifene suppressed the IL‐6‐induced STAT3 activation. Moreover, IL‐6 reduced the ratio of LC3‐Ⅱ/LC3‐Ⅰ, promoted P62 expression but Bazedoxifene reversed both changes in H9c2 cells. Our data suggested Bazedoxifene inhibited IL‐6/gp130 signalling and protected against cardiac remodelling together with function deterioration in TAC mice.

## 1 INTRODUCTION

Based on data from National Health and Nutrition Examination Survey (NHANES) 2011‐2014, 6.5 million American adults suffered heart failure (HF),^1^ of which the projected prevalence is expected to increase 46% from 2012 to 2030.^2^ While the risk factors of HF vary with geography, hypertension is considered to be highly associated with HF in almost all regions worldwide.^3^ Therefore, humans pay more attention to the prevalent magnitude of hypertensive heart disease (HHD). Its cardiovascular sequelae include myocardial hypertrophy, remodelling and the subsequent congestive HF. As a common feature seen in patients with hypertension or aortic stenosis, compensatory myocardial hypertrophy develops to restore the cardiac function in initial stage.^4^ However, pressure overload ultimately leads to HF characterized by cardiac dysfunction, structural remodelling and fibrosis in both interstitium and vasculature.^5^, ^6^, ^7^ Despite of significant advance in experimental research and clinical treatment to the spectrum of HHD, millions of patients still suffer from a gradual process of HF which accounts for the inordinate mortality.^8^ A wealth of information gives priority to angiotensin‐converting enzyme inhibitors (ACEIs)/angiotensin receptor blockers (ARBs) by a wide margin in the treatment of cardiac hypertrophy and heart failure but numerous patients are still not free from the cardiac disturbance. Gerber et al^9^ reported that from 2000 to 2010 the advance in improving survival kept levelling off and the overall 5‐year mortality of HF remained high at about 50% in Olmsted County, Minnesota. Compared with placebo, ARBs and direct renin inhibitors (DRIs) are not beneficial in decreasing the rate of efficacy outcomes including death, cardiovascular death, non‐fatal myocardial infarction, HF hospitalization and composite of cardiovascular death or HF hospitalization when HF therapy is not enough.^10^ Given the poor prognosis and the staggering social burden HHD and HF exert, a better understanding of the precise mechanism of HHD and HF merits accelerated investigation.^11^

Accumulating evidence suggested HHD is associated with inflammation. Levels of pro‐inflammatory cytokines have been found elevated in circulating blood in patients with heart failure.^5^ As one of the pro‐inflammatory cytokines, IL‐6 activates JAKs and STAT3 by recognizing its receptor (IL‐6R) and co‐receptor gp130, all of which play important roles in inflammation. IL‐6/gp130 signalling has been implicated in cardiovascular diseases.^12^, ^13^ There is ample evidence that IL‐6 is elevated during heart failure progression, which highlights IL‐6 as a potential target for intervention.^14^, ^15^ Studies have shown that infusion with IL‐6 leads to concentric cardiac hypertrophy and dysfunction in rats.^16^ The mRNA expression of IL‐6 in the myocardial tissues was elevated in TAC mice where abrupt pressure overload on left ventricle (LV) was produced.^17^, ^18^ Cardiac myocytes produce IL‐6 spontaneously, and persistent activation of gp130 is associated with cardiac hypertrophy in mice.^19^ However, the role of IL‐6 signalling is under debate due to different effects of IL‐6 inhibition on setting the trajectory for HHD progression. IL‐6 protects the heart from injury or does harm to myocardium depending on the kinetics of the host response. This cytokine could drive protective response in the short term in myocardial infarction but sustained elevated level of IL‐6 damages to the heart by resulting in chronic inflammation and fibrotic pathology.^20^ Several in vivo studies using IL‐6 knockout mice come to a divergence because both the negative^21^, ^22^ and positive^23^ effects of this genetic manipulation on the development of cardiac remodelling and dysfunction were observed. The existing discrepancies suggest a better understanding of the role of IL‐6/gp130 signalling in HHD is needed to elucidate whether regulation of this target could potentially affect the maladaptive cardiac development after an insult of pressure overload.

Previously our group reported that Bazedoxifene could inhibit phosphorylation of STAT3 by targeting IL‐6/gp130 interface in pancreatic cancer cells,^24^ which implied the potential effect of Bazedoxifene on IL‐6/gp130 signalling in cardiovascular diseases. Here, we found Bazedoxifene preserved cardiac function, and ameliorated myocardial remodelling caused by TAC in mice. Moreover, Bazedoxifene executed an inhibition of p‐STAT3 in H9c2 myoblasts stimulated with IL‐6 and in pressure overload mice at both the earlier and later stage. Moreover, Bazedoxifene might serve as another potent approach for manipulation of autophagy in vitro and in vivo.

## 2 MATERIALS AND METHODS

### 2.1 Animals and reagent

All animal experiments were performed in accordance with the Institutional Animal Use and Care Committee of Tongji Medical College, Tongji Hospital, Huazhong University of Science and Technology. Male wild‐type C57BL/6J mice (8 weeks old and ≈25 g) were purchased from the Jackson Laboratory. Mice were randomly assigned to three groups (n = 10 per group for long‐term observation and n = 5‐6 per group for acute window exploration): (a) sham group; (b) TAC group; (c) TAC+Bazedoxifene (hereinafter referred as BAZ) group. Murine TAC was performed to apply mechanical strain induced by pressure overload derived from aortic constriction on left ventricle as referenced.^25^ Mice were anaesthetized by 2% isoflurane mixed with 0.5‐1.0 L/min 100% O_2_. PE 90 tube was used to perform endotracheal intubation, then mechanical ventilation around 130 breaths/min and a tidal volume of 0.2‐0.3 mL was performed. Mice were placed in a supine position on a warming pad. Partial thoracotomy to the second rib was then performed using a chest retractor to retract sternum. The aortic arch was exposed and constricted by a 7‐0 silk ligature tied against a 27^1/2^ gauge blunt needle. The needle was removed resulting in a constriction with a diameter of about 0.36 mm. For Sham group, the same intervention without a tied knot was performed. For gavage Bazedoxifene (No. 15005, Cayman Chemical), stock solution was further diluted in phosphate buffer saline (PBS) containing 20% hydroxypropyl B cyclodextrin (HPBCD) (20 μg HPBCD/100 μL PBS, 0.2 mg/mL). In detail, the stock concentration of Bazedoxifene was 25 mg/mL in dimethylsulfoxide (DMSO). For intragastric gavage, the stock solution was further diluted in 0.2 mg/mL HPBCD to form mixed liquid containing 0.125 mg Bazedoxifene per 100 μL (5 μL DMSO, respectively) with regard to a mouse weighing 25 g. For Sham and TAC group, each mouse was daily administrated with mixed liquid comprised of 95 μL HPBCD (0.2 mg/mL) and 5 μL DMSO. Bazedoxifene (5 mg/kg) was administrated to mice of BAZ group via intragastric gavage once a day. All mice were given gavage from 3 days before surgery to the sacrifice at the indicated time‐points after surgery. In the long‐term observation, 3 mice of Sham group were sacrificed 4 weeks after surgery while 7 mice at 8 weeks. For TAC and BAZ groups, 4 mice were sacrificed in each group at 4 weeks after TAC and the left were all sacrificed at the time‐point of 8 weeks.

### 2.2 Histology and immunohistochemistry

Harvested hearts were embedded in paraffin or stored at −80°C before cutting. The embedded tissues were then sectioned and stained with haematoxylin‐eosin, picrosirius red or Massion's trichrome staining according to the manufacturer's protocols. The interstitial fields without any blood vessels and the perivascular fibrosis from the fields containing vasculature were imaged. For immunohistochemistry, we used antibody against IL‐6 (D220828, Sangon) to detect the protein expression in LV tissues according to the manufacturer's protocols. Image capture was operated using EVOS FL Auto Imaging System (Life Technologies, ThermoFisher Scientific).

### 2.3 Echocardiography

Echocardiograms were performed in East Hospital, Tongji University School of Medicine, Shanghai, China. Male wild‐type C57BL/6J mice (8 weeks old and ≈25 g) were randomly divided into three groups: (a) sham group, (b) TAC group, (c) TAC+BAZ (n = 4‐6 per group). The protocols for gavage was as described above. The echocardiographic parameters were assessed at the indicated time‐points and the potential side effect of BAZ on cardiac function was tested in n = 5 healthy mice (Methods S1). Mice were anaesthetized with isoflurane (3% for induction and 2% for maintenance). The gas was mixed in 1 L/min O_2_ and administrated via a facemask. Hair on the anterior chest was removed by chemicals and then discarded. All parameters were obtained by an experienced operator and the analysis of raw data was performed on a workstation installed with Vevo 2100 (FUJIFILM VisualSonics) by the same operator who was blind to all the experiment.

### 2.4 Cell culture and treatment

H9c2 cells were purchased from the American Type Culture Collection and maintained in Dulbecco's modified Eagle's medium (DMEM, KeyGEN BioTECH) high glucose supplemented with 10% foetal bovine serum (FBS, Gibco, ThermoFisher Scientific) and 1% penicillin/streptomycin (Sigma‐Aldrich). Cells were plated and cultured for 24 hours in the medium in a humidified 37°C incubator with 5% CO_2_. Prior to treatment H9c2 cells were serum starved for 8 hours. For Western blot experiments, starved H9c2 cells were stimulated by IL‐6 (PeproTech, No. 200‐06, 25 ng/mL) or angiotensin Ⅱ (AngⅡ, MedChemExpress, HY‐13948, 100 nmol/L) and harvested at the indicated time‐points. For the exploration of Bazedoxifene, starved cells were treated with Bazedoxifene (equivalent DMSO as control) for 2 hours followed by IL‐6 (25 ng/mL) or AngⅡ (100 nmol/L) induction for another 30 minutes. As to the experiments of soluble IL‐6 receptor (sIL‐6R), stimuli (IL‐6 or AngⅡ) and sIL‐6R were added to the supernatant culture medium of starved cells for 30 minutes, or administrated in the medium, respectively.

Cell treatment in the detection for the transcripts of hypertrophic markers and cell surface area was described as followed. H9c2 cells were planted in a 6‐well plate. Serum starved cells were pre‐treated with Bazedoxifene (20 μmol/L) for 1 hour, followed by treatment with IL‐6 (25 ng/mL) in the presence of Bazedoxifene for 1 hour. Then, H9c2 cells were cultured with medium containing IL‐6 (25 ng/mL) in the absence of Bazedoxifene for 48 hours. After washing with PBS, cells were collected for RNA extraction or fixed with 4% paraformaldehyde (Sigma‐Aldrich) for 20 minutes and stained with 0.5% crystal violet (ThermoFisher Scientific) for 30 minutes at room temperature. Excessive crystal violet was discarded, and image capture was operated using EVOS FL Auto Imaging System (Life Technologies, ThermoFisher Scientific) after the plates were dried. Cell surface area analysis was performed by ImageJ.

### 2.5 Quantitative PCR

Total RNA was extracted from frozen tissues and cultured cells using a Hipure Total RNA Mini kit (Magen) as the manufacturer's instructions. cDNA synthesis was performed using ReverTra Ace qPCR RT kit (TOYOBO Co., Ltd) following the manufacturer's instructions. A SYBR green PCR master mix kit (TOYOBO Co., Ltd) was used to perform real‐time quantitative reverse transcription polymerase chain reaction (qPCR) on StepOnePlus real‐time PCR system (Applied Biosystems, ThermoFisher Scientific). GAPDH was used as control and the 2^ΔΔCt^ method for data analysis. Primer sequences are listed as follows. mouse BNP: Forward CTGAAGGTGCTGTCCCAGAT, Reverse CCTTGGTCCTTCAAGAGCTG. mouse Col1A1: Forward TGAACGTGGTGTACAAGGTC, Reverse CCATCTTTACCAGGAGAACCAT. mouse Col3A1: Forward GCACAGCAGTCCAACGTAGA, Reverse TCTCCAAATGGGATCTCTGG. mouse Periostin: Forward AACCAAGGACCTGAAACACG, Reverse TGTGTCAGGACACGGTCAAT. mouse IL‐6: Forward CTCCCAACAGACCTGTCTATAC, Reverse CCATTGCACAACTCTTTTCTCA. mouse MMP2: Forward TGGCACCACCGAGGACTATGAC, Reverse ACACCACACCTTGCCATCGTTG. mouse GAPDH: Forward AGGTCGGTGTGAACGGATTTG, Reverse TGTAGACCATGTAGTTGAGGTCA. rat ANP: Forward GAGCGAGCAGACCGATGAAGC, Reverse TCCATCTCTCTGAGACGGGTTGAC. rat BNP: Forward AGCTCTCAAAGGACCAAGGC, Reverse TCTGCCCAAAGCAGCTTGAA. rat GAPDH: Forward GACATGCCGCCTGGAGAAAC, Reverse AGCCCAGGATGCCCTTTAGT.

### 2.6 Western blot

The pulverized cardiac tissues and the collected cultured cells were lysed in RIPA lysis buffer containing 1 mmol/L protease inhibitor and 1 mmol/L phosphatase inhibitor. The lysates were centrifuged at 13 800 *g* for 20 minutes at 4°C, and the supernatant was collected. The concentration of protein was determined by BCA protein assay kit. Equivalent amounts of protein were loaded and separated using 10%‐12% Bis‐Tris SDS‐PAGE gel electrophoresis, transferred to PVDF membrane and probed with antibodies. Antibodies against phospho‐STAT3 (Tyrosine 705, #9131, Cell Signaling Technology), phospho‐independent STAT3 (#4904, Cell Signaling Technology), LC3B (#3868, Cell Signaling Technology), P62/SQSTM1 (#18420‐1‐AP, Proteintech North America) and GAPDH (#10494‐1‐AP, Proteintech North America) were used. Horseradish peroxidase‐conjugated secondary antibodies and Immobilon Western Chemiluminescent HRP Substrate (AntGene Co., Ltd) were used for protein detection which was operated on ChemiDoc‐It 510 Imager with VisionWorks software (Ultra‐Violet Products Ltd) following the manufacturer's instructions.

### 2.7 Statistical analysis

Data were expressed as the means ± SEM from triplicated performed experiments. Comparison of multiple groups was analysed by one‐way analysis of variance (ANOVA) with Bonferroni's post hoc test. Statistical significance was defined as *P* < .05. All statistical analysis was performed with SPSS software (version 22.0). Quantitative assessment of Western blot and relative myocardial fibrosis area was performed by Image J.

## 3 RESULTS

### 3.1 Bazedoxifene attenuated cardiac hypertrophy induced by pressure overload in vivo

**Figure 1:**
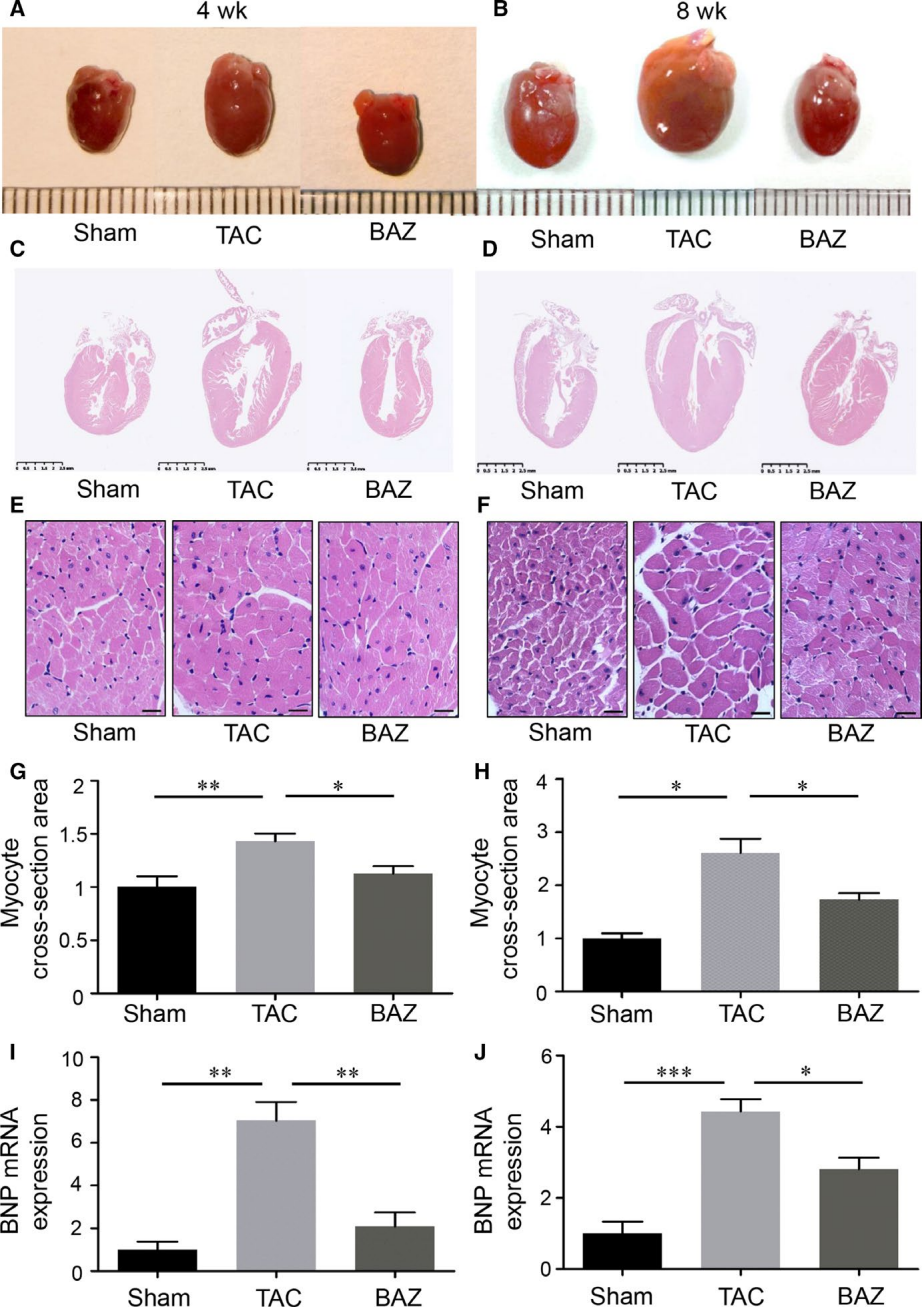
Morphological and hypertrophic molecular changes in heart tissues of mice. A, Representative images showing gross cardiac morphology of hearts from sacrificed mice after 4‐wk TAC. B, Gross cardiac morphology of hearts from sacrificed mice after 8‐wk TAC. C and D, Longitudinal sections of murine heart tissues stained with haematoxylin‐eosin at 4 (C) and 8 wk (D). E and F, Microscopic cross‐sections of 4‐ (E) and 8‐wk (F) TAC murine hearts stained with haematoxylin‐eosin and used for assessment of transverse cardiomyocyte area. G and H, Bar graphs showing the quantitative data for 4‐ (G) and 8‐week (H) transverse myocyte cross‐section area calculated by Image J. I and J, Bar graphs showing the mRNA expression of BNP in heart tissues from mice at 4 (I) and 8 wk (J). (n = 3 per group at 4 wk and n = 3‐4 per group at 8 wk) The relative abundance of mRNA expression or transverse myocyte area was quantified and normalized to GAPDH or that of Sham group. Data represent means ± SEM. The scale depicts 1mm for minimal interval in (A and B), 0.5mm for minimal interval in (C and D) and 20 μm in (E and F), respectively. *P < .05; **P < .01; ***P < .001

**Figure 2:**
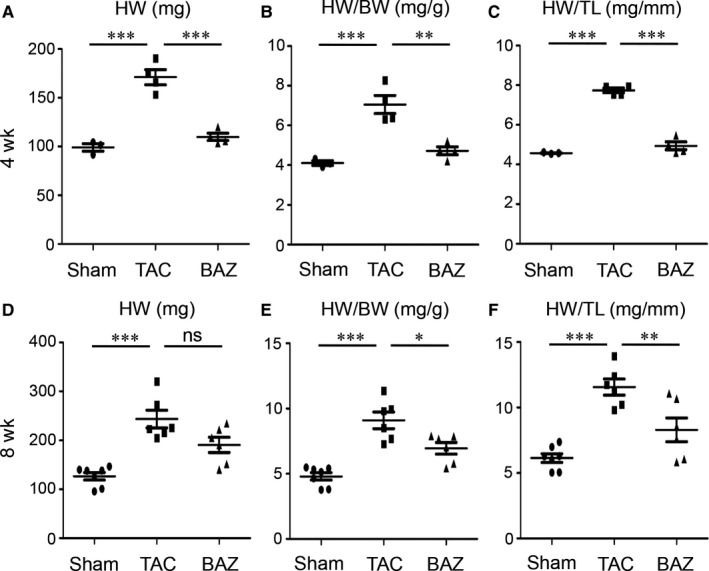
Calculation of HW, HW/BW and HW/TL of mice. A, Scatterplot showing the quantitative data for HW of mice at 4 wk. (n = 3‐4 per group) B, The ratio of HW/BW of mice at 4 wk. (n = 3‐4 per group) C, The ratio of HW/TL at 4 wk. (n = 3‐4 per group) D, HW of mice at 8 wk. (n = 6‐7 per group) E, The ratio of HW/BW of mice at 8 wk. (n = 6‐7 per group) F, The ratio of HW/TL at 8 wk. (n = 6‐7 per group) Scatterplot represents means ± SEM including individual data points. *P < .05; **P < .01; ***P < .001, “ns” stands for “None Significance”

To avoid potential bias resulting from different baseline, we chose age‐ and weight‐matched male mice for experiment. Heart tissues were harvested after 4 or 8 weeks of surgery. Gross morphology suggested the surgery of transverse aortic constriction increased the size of heart and Bazedoxifene attenuated this increase at 4 (Figure 1A,C) and 8 weeks (Figure 1B,D). We assessed the changes in heart weight (HW). The HW in TAC group (171.00 ± 15.57 mg) significantly increased (*P* < .001) in mice after 4 weeks of TAC manipulation compared with Sham counterparts (99.07 ± 6.71 mg). Intriguingly, the heart mass significantly decreased (*P* < .001) in BAZ group (109.83 ± 7.41 mg) (Figure 2A). As expected, the heart tissues of mice after 8 weeks of surgery showed a higher mass (TAC: 243.58 ± 44.26 mg vs Sham: 126.39 ± 20.29 mg, *P* < .001). However, the difference of HW between TAC and BAZ groups (190.53 ± 37.94 mg) at 8 weeks showed no statistical significance (Figure 2D). A consideration of importance is the fact that heart weight is relevant to the bodyweight (BW) of mice. So, we calculated the ratio of HW/BW. We found the ratio increased in mice after 4 and 8 weeks of TAC (TAC: 7.05 ± 0.92 vs Sham: 4.11 ± 0.20 at 4 weeks, *P* < .001, TAC: 9.10 ± 1.55 vs Sham: 4.80 ± 0.74 at 8 weeks, *P* < .001) while BAZ group exhibited lower ratio at both two time‐points (4.72 ± 0.41 at 4 weeks, *P* < .01, 6.95 ± 1.09 at 8 weeks, *P* < .05) (Figure 2B,E). We next used heart weight/tibia length (HW/TL) as another parameter to evaluate cardiac hypertrophy. As shown in figure, at 4 and 8 weeks the ratio of HW/TL in TAC mice was increased than that in Sham group (TAC: 7.74 ± 0.23 vs Sham: 4.57 ± 0.04 at 4 weeks, *P* < .001, TAC: 11.56 ± 1.50 vs Sham: 6.14 ± 0.88 at 8 weeks, *P* < .001) and the ratio in TAC mice was significantly higher compared with BAZ group (4.94 ± 0.39 at 4 weeks, *P* < .001, 8.30 ± 2.23 at 8 weeks, *P* < .01) (Figure 2C,F).

In addition, longitudinal sections of heart tissues from sacrificed mice after 4 and 8 weeks of TAC were prepared and assessment of myocyte cross‐sectional area was performed in each group. After aortic constriction, the transverse cardiomyocyte area was increased than that in Sham group at 4 (*P* < .01) and 8 weeks (*P* < .05). Bazedoxifene attenuated this increase significantly at both time‐points (Figure 1E‐H, *P* < .05 at 4 and 8 weeks). To confirm this TAC‐induced pathogenesis, we further explored the hypertrophic response at molecular level. It has been reported that brain natriuretic peptide (BNP) is recommended as one of the biomarkers for initial diagnostic tests, and it is suggested to be regarded as exclusion markers according to American Heart Association and European Society of Cardiology.^26^ We found the mRNA expression of BNP was increased by 4‐ (*P* < .01) and 8‐week TAC (*P* < .001) intervention. BAZ group showed a decreased expression level of BNP at both time‐points (Figure 1I and J, *P* < .01 at 4 weeks, *P* < .05 at 8 weeks). These results suggested administration of Bazedoxifene might alleviate cardiac hypertrophy caused by pressure overload in mice.

### 3.2 Bazedoxifene ameliorated pressure overload‐induced left ventricular dysfunction

**Figure 3:**
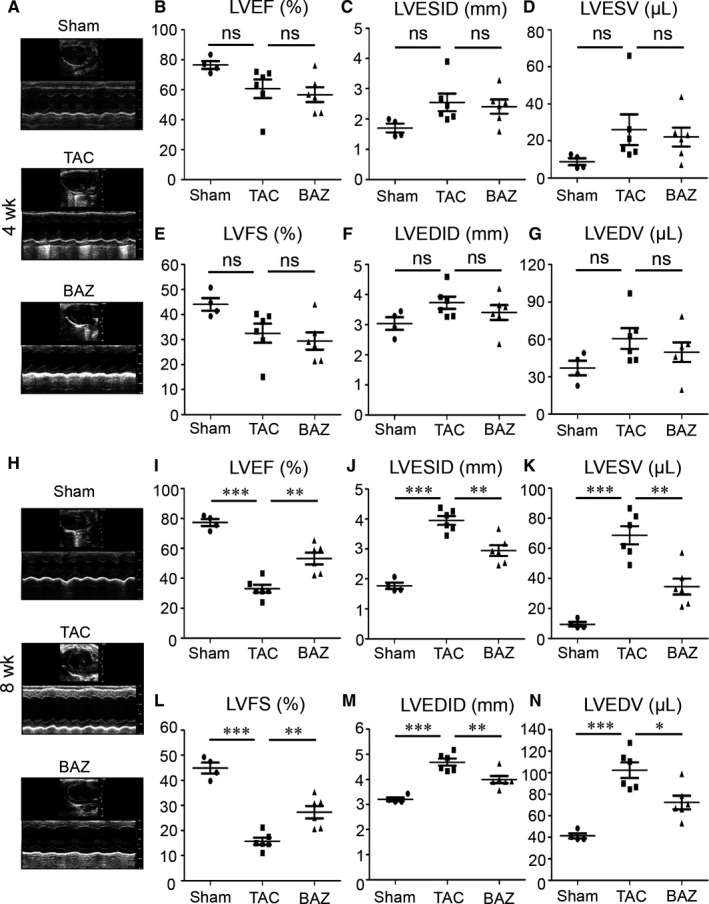
Echocardiographic profiles of mice at 4 and 8 wk. A and H, Representative images from mice at 4 and 8 wk by Vevo 2100 software. B‐G, LVEF, LVESID, LVESV, LVFS, LVEDID and LVEDV of mice were assessed at 4 wk after the surgery. I‐N, echocardiographic parameters of mice evaluated at 8 wk after the surgery. (n = 4‐6 per group) Scatterplot represents means ± SEM including individual data points. *P < .05; **P < .01; ***P < .001, “ns” stands for “None Significance”

Echocardiography was performed to assess the cardiac functional changes in mice after 4 weeks of TAC. Both left ventricular ejection fraction (LVEF, TAC: 60.71 ± 15.05% vs Sham: 76.54 ± 5.25%) and left ventricular fractional shortening (LVFS, TAC: 32.55 ± 9.43% vs Sham: 44.07 ± 5.00%) decreased in TAC group compared with the counterparts but with no significance. Bazedoxifene showed no significant benefits or exacerbation in preserving LVEF (56.72 ± 12.13%) and LVFS (29.43 ± 8.42%) at 4 weeks (Figure 3B,E). After 8 weeks of surgery mice in TAC group suffered LV dysfunction with a significant decrease of LVEF (TAC: 33.04 ± 6.42% vs Sham: 77.28 ± 4.70%, *P* < .001) and LVFS (TAC: 15.71 ± 3.41% vs Sham: 44.88 ± 4.31%, *P* < .001). Bazedoxifene dramatically ameliorated TAC‐induced cardiac dysfunction with better preserved LVEF (BAZ: 53.21 ± 9.59% vs TAC: 33.04 ± 6.42%, *P* < .01) and LVFS (BAZ: 27.30 ± 6.04% vs TAC: 15.71 ± 3.41%, *P* < .01) (Figure 3I,L). Consistently, the increased left ventricular end‐systolic internal diameter (LVESID) and volume (LVESV) in 8‐week TAC mice were less pronounced in BAZ group (BAZ: 2.95 ± 0.45 mm vs TAC: 3.95 ± 0.36 mm in LVESID, *P* < .01, BAZ: 34.52 ± 13.10 μL vs TAC: 68.70 ± 14.71 μL in LVESV, *P* < .01) (Figure 3J,K) which exhibited no significant protection at the time‐point of 4 weeks (BAZ: 2.41 ± 0.57 mm vs TAC: 2.55 ± 0.71 mm in LVESID, BAZ: 22.08 ± 12.50 μL vs TAC: 26.05 ± 20.22 μL in LVESV) (Figure 3C,D).

### 3.3 Cardiac remodelling and fibrosis induced by pressure overload was prevented by Bazedoxifene

Left ventricular end‐diastolic internal diameter (LVEDID) and volume (LVEDV) could be echocardiographic parameters of LV remodelling.^23^ There was no significant improvement on LVEDID and LVEDV of Bazedoxifene at 4 weeks (BAZ: 3.41 ± 0.60 mm vs TAC: 3.73 ± 0.50 mm in LVEDID, BAZ: 49.63 ± 19.28 μL vs TAC: 60.63 ± 20.29 μL in LVEDV) (Figure 3F,G) but mice given by gavage with Bazedoxifene exhibited smaller LVEDID and LVEDV with a significant difference compared with TAC mice after 8 weeks of surgery (BAZ: 3.99 ± 0.35 mm vs TAC: 4.68 ± 0.35 mm in LVEDID, *P* < .01, BAZ: 72.29 ± 15.35 μL vs TAC: 102.24 ± 17.73 μL in LVEDV, *P* < .05) (Figure 3M,N).

**Figure 4:**
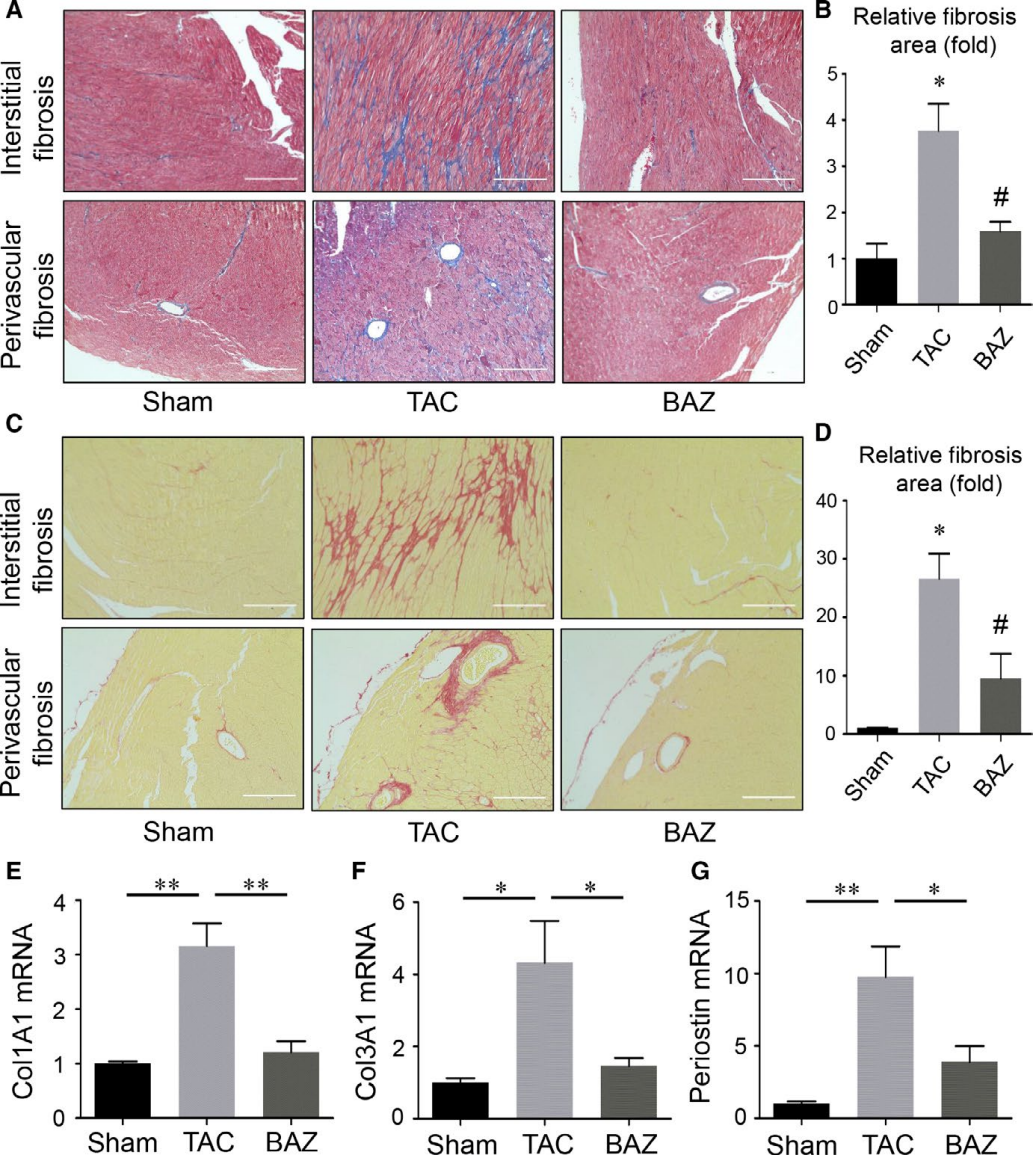
Bazedoxifene attenuated pressure overload‐induced LV fibrosis. LV tissues from mice after 8‐week surgery were sectioned and stained with Massion's trichrome (A and B) or picrosirius red (C and D). A‐D, scale bar, 200 μm, quantification of relative fibrosis area was performed by Image J, *P < .05 vs sham group; ^#^ *P* < .05 vs TAC group. E‐G, mRNA expression profiles of fibrosis‐related genes in murine hearts at 8 wk. The relative abundance of transcripts was quantified and normalized to GAPDH. (n = 3‐4 per group) Data represent means ± SEM. *P < .05; **P < .01

Cardiac remodelling was usually accompanied by fibrosis. We assessed LV myocardial interstitium and perivascular fibrosis by picrosirius red staining and Masson's staining. The characteristic of gradual pathogenesis that pressure overload features expects more prominent maladaptive remodelling with tissue scarring at the later time‐point. We found pressure overload increased both interstitial and perivascular fibrosis in LV myocardial tissues of mice heart after 8 weeks of TAC. Bazedoxifene could attenuate the TAC‐induced fibrosis (Figure 4A‐D, *P* < .05). Consistent with findings in histological observations above, the mRNA expression of periostin, collagen type Ⅰ alpha 1 and type Ⅲ alpha 1 (Col1A1 and Col3A1) measured in LV samples was found increased in mice after 8 weeks of TAC. Administration of Bazedoxifene could down‐regulate the mRNA expression of Col1A1, Col3A1 and periostin (Figure 4E‐G, *P* < .05), which indicated the pressure overload‐induced cardiac fibrosis might be ameliorated by Bazedoxifene. We also found the fibrosis already existed in myocardium at 4 weeks (Figure S1A‐D) when the cardiac function was disrupted but without significance. The mRNA expression of Col1A1, Col3A1 and periostin in heart tissues at 4 weeks were shown (Figure S1E‐G). Although the differences in some parameters among three groups were not that evident, TAC promoted cardiac fibrosis and Bazedoxifene treatment tended to ameliorate this pathology.

### 3.4 Inhibition of Bazedoxifene on IL‐6 signalling pathway in heart tissues of pressure overload mice

**Figure 5:**
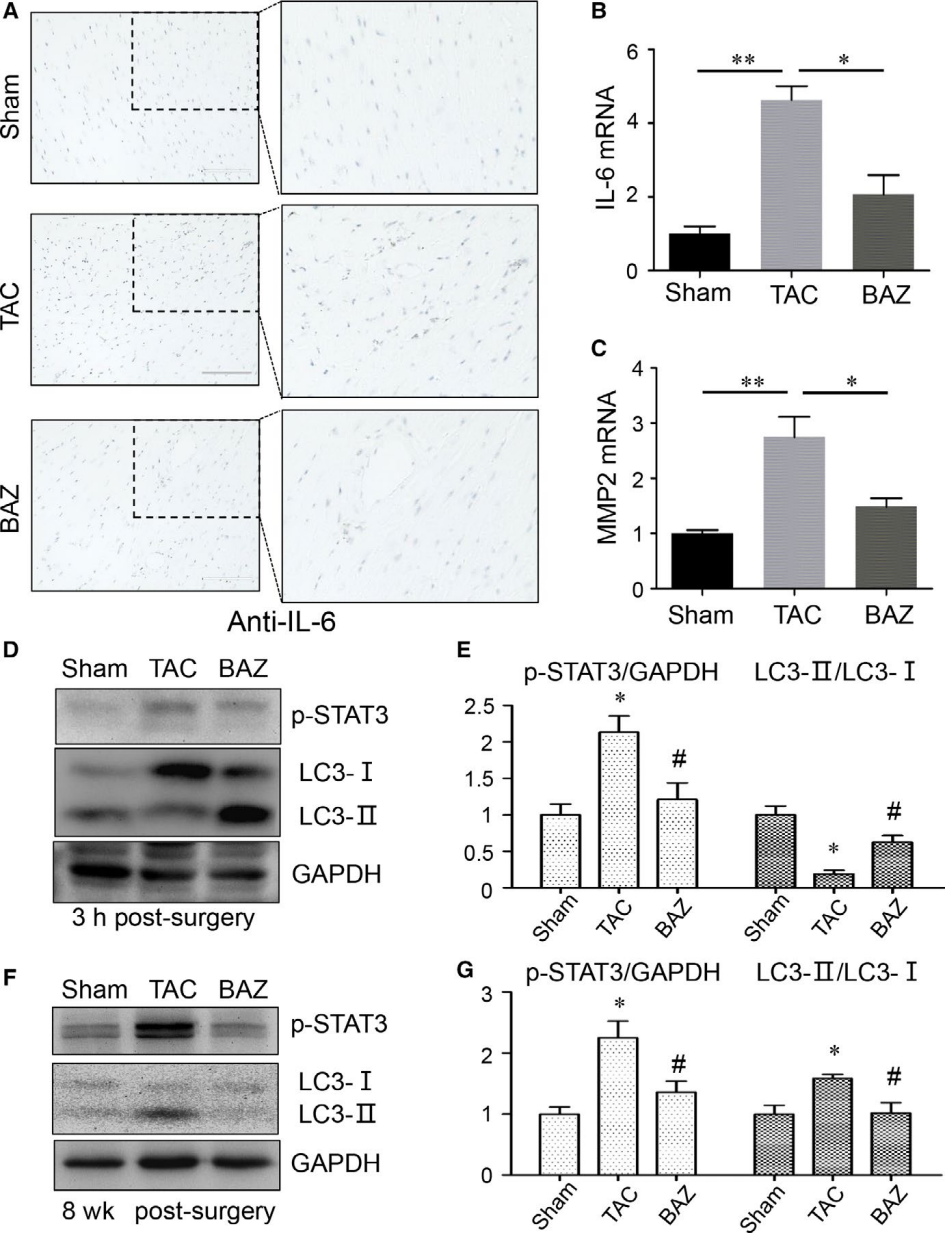
A, Representative images of immunohistochemical staining of IL‐6 (brown blots) in heart tissues of mice at 8 wk. The scale represents 100μm (left panel). Amplified images of the box depicting the staining of protein of interest (right panel). B, Bar graphs showing the mRNA expression of IL‐6 in heart tissues from mice at 8 wk. (n = 3‐4 per group) C, The mRNA expression of MMP2 in murine hearts at 8 wk. (n = 3‐4 per group) The relative abundance of transcripts was quantified and normalized to GAPDH. (*P < .05; **P < .01.) D and E, Western blots with quantification showing the expression of p‐STAT3 and LC3 in hearts of mice 3 h after surgery (n = 5‐6 per group). F and G, Western blots with quantification showing p‐STAT3 and LC3 expression in murine hearts 8 wk post‐TAC (n = 3‐4 per group). The relative expression of p‐STAT3 was quantified and normalized to that of GAPDH. The conversion of LC3 was quantified by the ratio of LC3‐Ⅱ/LC3‐Ⅰ. *P < .05 vs sham group; ^#^ *P* < .05 vs TAC group. Data represent means ± SEM

Huameng Li et al^24^ found Bazedoxifene could inhibit IL‐6 signalling in cancer cells. We next detected the effect of Bazedoxifene on IL‐6 signalling in heart tissues of mice. As shown in Figure 5, Bazedoxifene decreased the expression of IL‐6 in the harvested heart tissues of 8‐week TAC mice by immunohistochemical staining (Figure 5A). The mRNA expression of IL‐6 and MMP2 was elevated (*P* < .01) in myocardial tissues of 8‐week TAC mice but decreased by Bazedoxifene (*P* < .05) (Figure 5B,C). We also paid attention to the acute window after surgery and the long‐term effect in mice. We found an inhibition of Bazedoxifene on TAC‐induced p‐STAT3 overexpression in heart tissues at 3 hours (Figure 5D,E, *P* < .05) and 8 weeks (Figure 5F,G, *P* < .05) after surgery. Moreover, we demonstrated increased ratio of LC3‐Ⅱ/LC3‐Ⅰ in heart tissues by Bazedoxifene compared with that in TAC group in initial stage of pathogenesis. (Figure 5D,E, *P* < .05) However, 8‐week TAC up‐regulated the ratio of LC3‐Ⅱ/LC3‐Ⅰ in heart and durative Bazedoxifene administration reduced this change (Figure 5F,G, *P* < .05). We next explored the potential mechanisms in H9c2 cells.

### 3.5 The effect of Bazedoxifene on IL‐6 signalling and autophagic markers in H9c2 myoblasts

**Figure 6:**
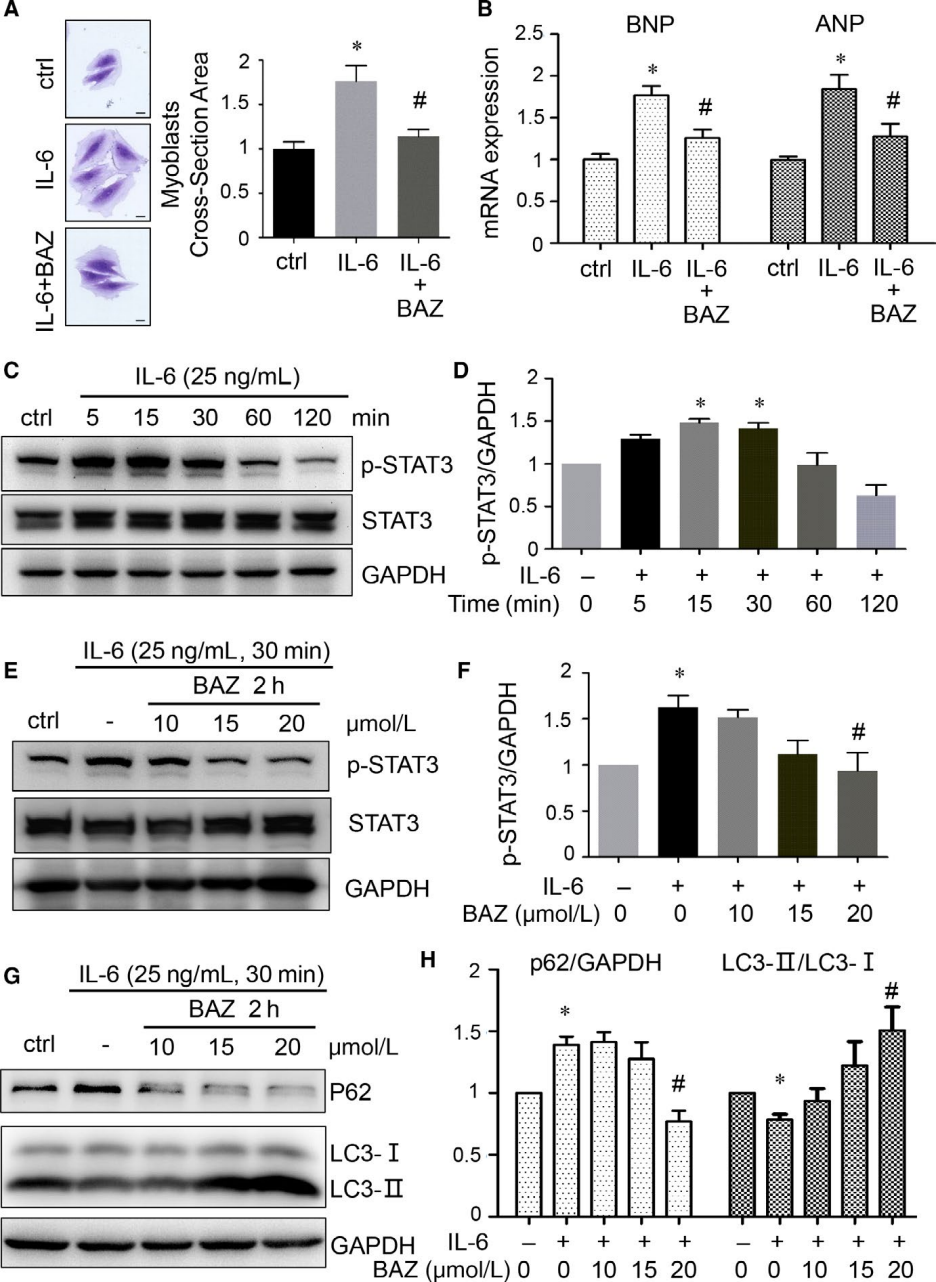
A and B, Bazedoxifene attenuated IL‐6‐stimulated cell hypertrophy in H9c2 myoblasts (A, scale bar, 20 μm). Bazedoxifene down‐regulated the transcripts of ANP and BNP induced by IL‐6 (B, n = 4). *P < .05 vs ctrl group; ^#^ *P* < .05 vs IL‐6 group without Bazedoxifene. C and D, Western blots with quantification showing the expression of p‐STAT3 in H9c2 cells stimulated by IL‐6 of 25 ng/mL at serial time‐points as indicated. E and F, after serum starvation H9c2 cells were treated without or with Bazedoxifene (10, 15, 20 μmol/L) for 2 h and then administrated with IL‐6 (25 ng/mL) in presence of Bazedoxifene for another 30 min. Bazedoxifene inhibited IL‐6‐induced STAT3 activation. G and H, Treated without or with Bazedoxifene (10, 15, 20 μmol/L) for 2 h, H9c2 cells were stimulated by IL‐6 (25 ng/mL) for another 30 min. IL‐6 trigger decreased ratio of LC3‐Ⅱ/LC3‐Ⅰ and promoted P62 expression. Bazedoxifene increased the ratio and down‐regulated expression of P62 in H9c2 cells. Quantitative data of p‐STAT3 or P62 were normalized to those of GAPDH. The conversion of LC3 was quantified by the ratio of LC3‐Ⅱ/LC3‐Ⅰ. Data of control group were set arbitrarily as 1. Data represent means ± SEM. *P < .05 vs ctrl group; ^#^ *P* < .05 vs IL‐6 group without Bazedoxifene

IL‐6 led to cell hypertrophy in H9c2 cells and treatment of Bazedoxifene in initial stage of IL‐6 stimuli alleviated this cellular response to IL‐6 (Figure 6A, *P* < .05). In the study by Zhao et al,^23^ the authors showed IL‐6 trigger resulted in an enlargement in size of H9c2 cells while WP1066 (a STAT3 inhibitor) decreased the cell size. We further detected two hypertrophic markers, atrial natriuretic peptide (ANP) and BNP. We found IL‐6 significantly increased the mRNA expression of two markers (*P* < .05). Bazedoxifene could down‐regulate IL‐6‐induced mRNA level of ANP and BNP in vitro (Figure 6B, *P* < .05). In the cultured H9c2 myoblasts IL‐6 (25 ng/mL) induced the phosphorylation of STAT3 (Figure 6C,D, *P* < .05 at 15 and 30 minutes). We then explored the effect of Bazedoxifene on IL‐6 signalling. The IL‐6‐triggered enhanced phosphorylation of STAT3 (p‐STAT3) was inhibited by Bazedoxifene. (Figure 6E,F, *P* < .05 at 20 μmol/L).

Microtubule‐associated protein 1 light chain 3 (LC3) is one of the autophagic markers and the role for P62 has been established by many studies in protein turnover through autophagy. We demonstrated the decreased ratio of LC3‐Ⅱ/LC3‐Ⅰ in heart tissues harvested from the mice after acute pressure overload trigger while the ratio in BAZ mice was higher (Figure 5D, *P* < .05). We further noticed the reduced ratio of LC3‐Ⅱ/LC3‐Ⅰ and increased expression of P62 with IL‐6 treatment compared to those in H9c2 cells as control. Bazedoxifene preserved the ratio and suppressed the expression of P62 (Figure 6G,H, *P* < .05 at 20 μmol/L).

### 3.6 IL‐6 contributed to the response to Angiotensin Ⅱ in H9c2 myoblasts

**Figure 7:**
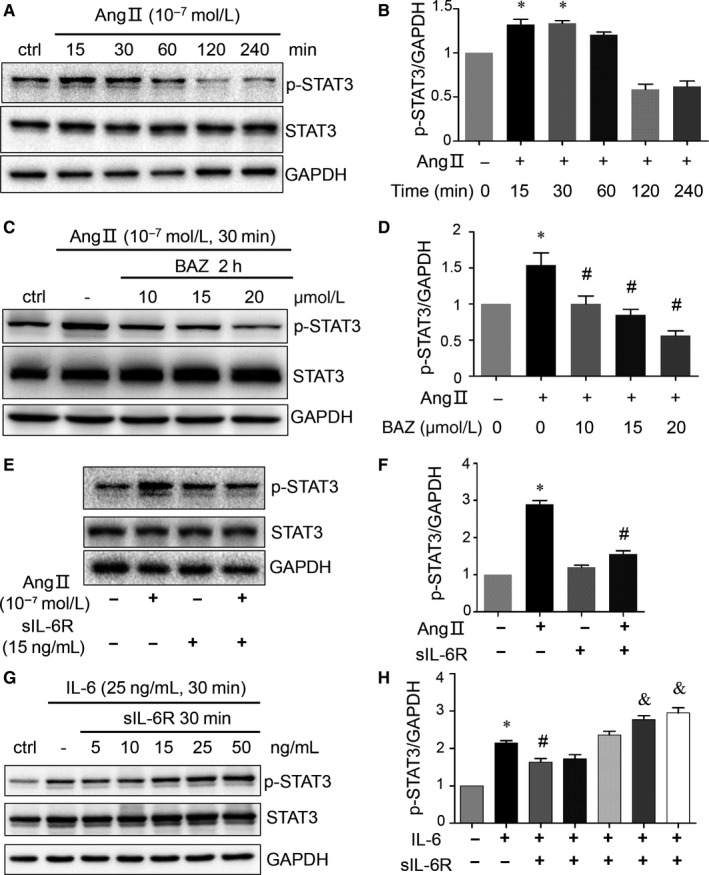
IL‐6 contributed to the response to AngⅡ in H9c2 cells. A and B, Western blots with quantification showing the expression of p‐STAT3 in H9c2 cells stimulated by AngⅡ of 100 nmol/L at serial time‐points as indicated. C and D, after serum starvation H9c2 cells were treated without or with Bazedoxifene (10, 15, 20 μmol/L) for 2 h and then administrated with AngⅡ (100 nmol/L) in presence of Bazedoxifene for another 30 min. Bazedoxifene suppressed AngⅡ‐induced STAT3 activation. (For B and D, *P < .05 vs ctrl group; ^#^ *P* < .05 vs AngⅡ group without Bazedoxifene) E and F, Inhibition of AngⅡ‐induced p‐STAT3 by sIL‐6R. Cells were cultured with AngⅡ (100 nmol/L) and sIL‐6R (15 ng/mL) simultaneously for 30 min as indicated. G and H, Effect of sIL‐6R of different concentration on IL‐6 signalling transduction. H9c2 cells were cultured with IL‐6 (25 ng/mL) and sIL‐6R (5, 10, 15, 25, 50 ng/mL) simultaneously for 30 min as indicated. Quantitative data of p‐STAT3 were normalized to those of GAPDH. Data of control group were set arbitrarily as 1. Data represent means ± SEM. (For F and H, *P < .05 vs ctrl group; ^#^ *P* < .05 vs AngⅡ or IL‐6 group without sIL‐6R; ^&^ *P* < .05 vs IL‐6 group without sIL‐6R)

We also found AngⅡ induced the phosphorylation of STAT3 (Figure 7A,B, *P* < .05 at 15 and 30 minutes), and this induction could be inhibited by Bazedoxifene in a concentration dependent manner (Figure 7C,D, *P* < .05), which indicated that IL‐6 might contribute to the response to AngⅡ in H9c2 cells. Moreover, the AngⅡ‐induced p‐STAT3 could be inhibited by soluble IL‐6 receptor (sIL‐6R) (Figure 7E,F, *P* < .05), a specific constituent of trans‐signalling of IL‐6, which is considered limiting of IL‐6‐induced p‐STAT3 at low concentrations but permissive of p‐STAT3 at higher concentrations (Figure 7G,H, *P* < .05 at 5, 25 and 50 ng/ml), indicating IL‐6 might play an important role in response to AngⅡ in H9c2 myoblasts.

## 4 DISCUSSION

As people attain an advanced age, HHD becomes more responsible for the retained mortality of cardiac disorders. The poor clinical outcomes reveal that the prospects for pharmacological intervention of HHD require a basis of more elucidated mechanisms.

Various signalling pathways contributing to HHD‐associated LV remodelling and dysfunction have been annotated. Studies suggested the disease triggers of hypertrophic programme could be ascribed to the over‐activation of renin‐angiotensin‐system (RAS).^27^, ^28^, ^29^ Interestingly, Harada et al^30^ found long‐term TAC still resulted in cardiac hypertrophy in angiotensin Ⅱ type 1A receptor knockout mice. In TAC mice captopril alleviated cardiac remodelling only when administrated during sleep^31^ while Olmesartan, Candesartan and Losartan, three of ARBs, conferred benefits in TAC‐induced cardiac injury even in angiotensinogen‐knockdown mice,^32^ all of which highlighted uncovered mechanism beside AngⅡ signalling pathway and the large gap in the understanding of RAS and AngⅡ in HHD. The specific role of IL‐6/gp130 signalling in HHD still needs more studies to elucidate. In addition to the pro‐hypertrophic role of IL‐6 in rats, IL‐6 deficiency was found to attenuate AngⅡ induced cardiac pathology^33^, ^34^ and excessive activation of gp130 induces myocardial hypertrophy in mice.^19^ In contrary to the notion that IL‐6 might be pro‐hypertrophic and fibrotic, Kaminski et al and Lai et al^21^, ^22^ considered IL‐6 as non‐essential in cardiac hypertrophy using IL‐6 knockout mice. The divergence ascribed to the results of present studies makes the role of IL‐6 in the development of pressure overload‐induced cardiac hypertrophy and HF in mice complicated.

IL‐6 not only recognizes IL‐6R and gp130 to transduce signalling (classical pathway) but also binds to sIL‐6R to activate the downstream molecular (trans‐signalling pathway) independent of membrane IL‐6R. Both pathways contribute to the pleiotropic effect of IL‐6. Recently, Zhao et al concluded that IL‐6 played a central role in cardiac hypertrophy because of the benefits in TAC mice through IL‐6 depletion resulting in inhibited p‐STAT3 in hearts. Moreover, their conclusion suggested the necessity of IL‐6 in mediation of hypertrophic response induced by AngⅡ and phenylephrine (PE) in vitro.^23^ As shown in our results section, AngⅡ elicited STAT3 phosphorylation which could be decreased by Bazedoxifene. The suppressive effect of sIL‐6R on AngⅡ‐induced p‐STAT3 provided another evidence which stood for the potential role of IL‐6 in response to AngⅡ: the hypertrophic effect of AngⅡ might be partly mediated by IL‐6.

Given the evidence that AngⅡ is considered as a crucial mediator of HHD^27^, ^28^, ^29^ and is the target of developed therapeutic manipulation in wide range of cardiovascular diseases, we further performed TAC mice model to explore the effect of Bazedoxifene on cardiac pathological changes. As a tool to explore cardiac hypertrophy, remodelling and the consequent heart failure, experimental TAC is performed to produce pressure overload on LV to mimic HHD. The results in the study by Lai et al^22^ showed no significant effect of deletion of IL‐6 on LV remodelling and dysfunction after 2‐ and 4‐week TAC but Zhao et al^23^ reported that deletion of IL‐6 ameliorates left ventricular hypertrophy, fibrosis and dysfunction in mice with a longer follow‐up (6 weeks) after TAC. In terms of the differences in prior researches, the duration of follow‐up in our study was set 8 weeks after the primary surgery. Meanwhile, all male mice were age and weight matched to achieve a better normalized baseline. We found that TAC did not significantly altered cardiac function and Bazedoxifene exerted no significant protective effect on cardiac function at 4 weeks after aorta ligation. The benefits of Bazedoxifene were pronounced as evidenced by the 8‐week observations. DeAlmeida et al^35^ has reported the TAC mice operated using a 27^1/2^ gauge needle are expected to develop cardiac hypertrophy within 1‐2 weeks and cardiac dilation after 6‐8 weeks. Heart of TAC mice has to pump more than normal, and can determine a transient increase in the cardiac contraction, which might be erroneously recorded as an amelioration of his function. Prolonged pressure overload in the long term leads to a gradual maladaptive remodelling, with LV dilatation, tissue scarring and heart failure, which appears evident at 6‐8 weeks after surgery.^35^ We found that TAC and BAZ had effects on heart mass as well as cardiac hypertrophy already at 4 weeks. The compensatory cardiac hypertrophy at early stage of TAC‐induced pathology might account for the reason why TAC negatively effects and Bazedoxifene shows cardioprotective effect on heart function only significantly at 8 weeks after surgery in our study, which suggested that although the pharmacological intervention and insult all started in the initial phases, the redefined trajectory of disease progression might be identified in a long‐term period. Intriguingly, Furihata et al^36^ found operation with a 0.400 mm gauge needle led to the state of hypertrophy without left ventricular dysfunction at 4 weeks after surgery, in which the authors also showed that the 0.385 mm and 0.375 mm group (smaller constriction diameter than that in our study) of mice represented efficient in left ventricular failure at 4 weeks, while the average bodyweight of mice (19‐26 g) in the research was lower than that in our study. In another study Richards et al^37^ reported that TAC mice using a 27 gauge blunt needle (smaller constriction diameter than 0.400 mm) developed significant cardiac dysfunction, however, the important point of note they found was the cardiac response to different degrees of constriction seemed not to be quite consistent with the severities of phenotype because the exacerbation of several cardiac parameters was not step‐wise such as fractional shortening, ejection fraction, left atrial area, etc These reports indicate the underlying mechanisms governing these distinct responses to TAC in murine heart under different degrees of insult are still not clear, which deserves further study to figure out the potential reasons.

The underlying mechanisms that involved with IL‐6 signalling in HHD were yet to be disambiguated because of the complicated networks comprised of multiple biological process. It has been reported that the expression of IL‐6 is in positive correlation with that of MMP2 in several types of cardiovascular diseases such as aortic dissection,^38^ lipopolysaccharide‐induced cardiac fibrosis^39^ and pressure overload‐associated cardiac extracellular matrix (ECM) remodelling.^40^, ^41^ In addition to the fact that an IL‐6 dependent increase of MMP2 and a MMP2‐dependent increase in IL‐6 are observed,^42^, ^43^, ^44^ STAT3 directly binds to the promoter of MMP2 to regulate cellular biological process.^45^ IL‐6 activates STAT3 phosphorylation and promotes the expression of MMP2. Activated MMP2 degrades the ECM rendering aggravated remodelling with severe inflammation, which might lead to a IL‐6‐MMP2 feedback contributing to an uncontrolled response. Therefore, the activity of IL‐6/STAT3/MMP2 axis could be related to the severity of cardiac remodelling where inflammation plays a role of importance. We found increased expression of phosphorylation of STAT3 in LV tissues of mice after 3‐hour TAC. Although no discerned differences of heart size were identified (data not shown), the activity of IL‐6 signalling pathway might have been modulated in acute window after TAC triggers and then continuously be over‐activated for a long time. Moreover, study has shown that inhibition of STAT3 signalling by either AG490 or STAT3 siRNA results in a decrease in both transcription and secretion of IL‐6 in human vascular endothelial cells.^46^ In our study, Bazedoxifene led to decreased activation of STAT3 and down‐regulated mRNA expression of MMP2 at the later time‐point, which might result in a mitigated heart condition rendering down‐regulated IL‐6 expression as shown in Figure 5A. Additionally, another study shows IL‐6‐induced STAT3 phosphorylation also promotes the transcription of STAT3, which leads to the accumulation of unphosphorylated STAT3 (u‐STAT3) allowing the crosstalk of STAT3 and NF‐κB to propagate and amplify the inflammatory response.^47^ We did not demonstrate whether this additional transcriptional mechanism involved in our study but it is worth being studied in future. Further in study by Zhao et al,^23^ WP1066, a STAT3 inhibitor, has been documented to attenuate the cell surface area in H9c2 cells in presence of IL‐6 but with no use in mice. We found Bazedoxifene decreased cell surface area and myocytes area in IL‐6 stimulated H9c2 cells and TAC mice the balance between the maladaptive response and the reparative compensation defines the progression of HHD. TAC‐induced pressure overload triggers pathological imbalance promoting to create a new baseline. Therefore, exogenous treatment of Bazedoxifene might contribute to reversing the altered balance attempting to result in better prognosis. Moreover, our pharmacological intervention by Bazedoxifene is more translatable to advanced therapy of human disease compared with genome manipulation like IL‐6 deletion.

Autophagy is the process involving the selection and degradation of cellular components under orchestrated regulation. Matured autophagosome containing sequestrated cellular cargos tagged for degradation requires the fusion with a lysosome to form the autolysosome where the contents are degraded into the constituent build blocks.^48^ During autophagosome formation, the nascent LC3 proceeds to become LC3‐Ⅰ with latent cytosolic distribution. Lipidation of LC3 is the conversion of cytosolic LC3‐Ⅰ to LC3‐Ⅱ which is incorporated into the double‐membrane structure of autophagosomes and mediates protein incorporation. Therefore, the expression of LC3‐Ⅱ/LC3‐Ⅰ could be used to evaluate the abundance of autophagosomes.^49^, ^50^ P62, known as one of autophagy receptors, harbours both ubiquitin‐binding domains and LC3‐interacting region conferring the selectivity to cargos for autophagic clearance. However, accumulation of P62 could account for the markedly slowed proteasomal degradation by binding ubiquitinated proteins.^51^ Studies found decreased markers of autophagy during an initial (1 week) phase in TAC mice compared with the counterparts^52^ and excessive P62 expression is found in aberrant protein aggregates.^53^ An opinion suggests that cardiac autophagy at baseline level or short‐term activation primes the myocardium to withstand overload pressure but excessive and sustained autophagy might contribute to cardiac malfunction under a hypertrophic insult.^50^ Induction of autophagy is considered beneficial in the setting of acute energy stress including fasting, nutrient deprivation and ischaemia‐reperfusion.^54^, ^55^, ^56^ Our results suggested treatment of Bazedoxifene might result in preserved ratio of LC3‐Ⅱ/LC3‐Ⅰ and down‐regulated expression of P62 under an acute insult of IL‐6 (30min) in H9c2 cells, which might indicate an enhanced autophagic flux. In animal study, Bazedoxifene increased this ratio at 3 hours after TAC but attenuated the aberrant up‐regulation of LC3‐Ⅱ/LC3‐Ⅰ at 8 weeks post‐TAC. In general, Bazedoxifene alleviated over‐activation of STAT3 and moderated the aberrant autophagic process caused by pressure overload at both two time‐points. This special effect of Bazedoxifene in our results might confer benefit in protein turnover by enhanced degradation of maladaptive cellular components in response to acute stress while mitigating over‐activation of autophagy in the long term, which accounted for the potential mechanisms underlying the benefit of Bazedoxifene intake in TAC mice in part.

We found the induction of p‐STAT3 was coincident with reduction of ratio of LC3‐Ⅱ/LC3‐Ⅰ in IL‐6 stimulated H9c2 cells and in TAC mice at the earlier stage, but at the later stage in TAC mice up‐regulation of p‐STAT3 was accompanied by increased ratio of LC3‐Ⅱ/LC3‐Ⅰ. A review about the understanding of STAT3 in autophagy summarized that different modified or localized STAT3 exhibited pro‐ or anti‐autophagy function in transcription‐dependent and transcription‐independent manner in various circumstances.^57^ Inhibition of STAT3 could lead to induction of lipidation of LC3A resulting in up‐regulation of autophagy in glioma cells.^58^ However, the mechanisms underlying how STAT3 plays context‐dependent role in autophagy is yet to be elucidated. Further studies are required to discern the potential interaction between IL‐6 signalling pathway and LC3 or P62 in the nuanced autophagy throughput in pressure overload‐induced perturbation.

Recently, another study found circulating IL‐6 and tumour necrosis factor alpha (TNF‐α) instead of pressure load are associated with concentric left ventricular remodelling independent of left ventricular mass,^59^ which indicated we should take more efforts to explore other potential roles of IL‐6 as well as inflammation in cardiovascular diseases. The inhibition of IL‐6 signalling by Bazedoxifene and the benefits it conferred in murine pressure overload pathology might provide evidence for the potential role of Bazedoxifene in anti‐inflammatory therapy in HHD and even other cardiovascular diseases. Moreover, no anti‐inflammatory therapies are approved for the clinical treatment of HHD and its sequelae to date. Bazedoxifene has been approved by US Food and Drug Administration (FDA) for the treatment of osteoporosis in post‐menopausal women with high risk of breast cancer, which provides the advantages of Bazedoxifene in clinic use and we also found Bazedoxifene had no significant effect on LVEF, LVFS, LVESID, LVEDID, LVESV and LVEDV in healthy C57BL/6J mice (Figure S2A‐F). Future studies might shed new insights into the promising effect of Bazedoxifene, and our discovery might supply the development of novel avenues in treatment of cardiovascular diseases with inspiration.

Collectively, we demonstrated Bazedoxifene suppressed the activity of IL‐6 signalling pathway and regulated autophagy in vitro and in vivo. Bazedoxifene intake could decrease the hypertrophic markers in vivo and in vitro, and attenuate TAC‐induced cardiac remodelling with down‐regulated fibrotic markers. However, the existing gap in our knowledge base in the field of HHD should be noted. The pathology that murine model mimics differs from HHD in humans. Studies have proved that Bazedoxifene inhibits IL‐6/gp130 signalling pathway in several types of cancer,^60^, ^61^, ^62^ but whether the observed benefit of Bazedoxifene intake is dependent of IL‐6 signalling in pressure overload mice has not been established. Notably, the 3‐hour to 8‐week results suggested the sustained STAT3 activation and dynamic alteration of LC3 conversion might participate throughout the whole follow‐up, which indicated the potential requirement of durative Bazedoxifene administration, while the mechanisms underlying the adverse effect of Bazedoxifene on LC3‐II/LC3‐I in acute window and the long term need further study to clarify. To answer the question about what kinds of roles the immune cells and inflammation play in HHD also requires an in‐depth understanding of the potential implication different cells have, which will aid in the interpretation of controversial studies at present.

## CONFLICT OF INTEREST

The authors confirm that there are no conflicts of interest.

## AUTHOR CONTRIBUTIONS

Li Lin and Jiagao Lv designed research, Wei Shi and Junyi Guo analysed data, Wei Shi, Haiyan Ma, Tianshu Liu, Dan Yan, Pengcheng Luo, Jingwen Tao and Shengqi Huo performed research, Wei Shi and Maocai Zhai wrote the paper, Chenglong Li, Jiayuh Lin, Cuntai Zhang and Sheng Li contributed analytic tools.

## Supporting information

Supplementary Material

Method S1

## Data Availability

The data that support the findings of this study are available from the corresponding author upon reasonable request.

## References

[1] Benjamin EJ , Virani SS , Callaway CW , et al. Heart disease and stroke statistics‐2018 update: a report from the American Heart Association. Circulation. 2018;137:e67‐e492. 29386200 10.1161/CIR.0000000000000558

[2] Heidenreich PA , Albert NM , Allen LA , et al. Forecasting the impact of heart failure in the United States: a policy statement from the American Heart Association. Circ Heart Fail. 2013;6:606‐619. 23616602 10.1161/HHF.0b013e318291329a PMC3908895

[3] Khatibzadeh S , Farzadfar F , Oliver J , et al. Worldwide risk factors for heart failure: a systematic review and pooled analysis. Int J Cardiol. 2013;168:1186‐1194. 23201083 10.1016/j.ijcard.2012.11.065 PMC3594565

[4] Lyon RC , Zanella F , Omens JH , Sheikh F . Mechanotransduction in cardiac hypertrophy and failure. Circ Res. 2015;116:1462‐1476. 25858069 10.1161/CIRCRESAHA.116.304937 PMC4394185

[5] Torre‐Amione G . Immune activation in chronic heart failure. Am J Cardiol. 2005;95:3C‐8C; discussion 38C–40C. 10.1016/j.amjcard.2005.03.006 15925558

[6] Thum T , Galuppo P , Wolf C , et al. MicroRNAs in the human heart: a clue to fetal gene reprogramming in heart failure. Circulation. 2007;116:258‐267. 17606841 10.1161/CIRCULATIONAHA.107.687947

[7] Shimizu I , Minamino T . Physiological and pathological cardiac hypertrophy. J Mol Cell Cardiol. 2016;97:245‐262. 27262674 10.1016/j.yjmcc.2016.06.001

[8] Schefold JC , Filippatos G , Hasenfuss G , et al. Heart failure and kidney dysfunction: epidemiology, mechanisms and management. Nat Rev Nephrol. 2016;12:610‐623. 27573728 10.1038/nrneph.2016.113

[9] Gerber Y , Weston SA , Redfield MM , et al. A contemporary appraisal of the heart failure epidemic in Olmsted County, Minnesota, 2000 to 2010. JAMA Intern Med. 2015;175:996‐1004. 25895156 10.1001/jamainternmed.2015.0924 PMC4451405

[10] Bangalore S , Kumar S , Messerli FH . When conventional heart failure therapy is not enough: angiotensin receptor blocker, direct renin inhibitor, or aldosterone antagonist? Congestive Heart Fail. 2013;19:107‐115. 10.1111/chf.12011 23241032

[11] Dick SA , Epelman S . Chronic heart failure and inflammation: what do we really know? Circ Res. 2016;119:159‐176. 27340274 10.1161/CIRCRESAHA.116.308030

[12] Hunter CA , Jones SA . IL‐6 as a keystone cytokine in health and disease. Nat Immunol. 2015;16:448‐457. 25898198 10.1038/ni.3153

[13] Kukielka GL , Smith CW , Manning AM , et al. Induction of interleukin‐6 synthesis in the myocardium. Potential role in postreperfusion inflammatory injury. Circulation. 1995;92:1866‐1875. 7671371 10.1161/01.cir.92.7.1866

[14] Zhou L , Miao K , Yin B , et al. Cardioprotective Role of Myeloid‐Derived Suppressor Cells in Heart Failure. Circulation. 2018;138:181‐197. 29437117 10.1161/CIRCULATIONAHA.117.030811

[15] Mann DL . Innate immunity and the failing heart: the cytokine hypothesis revisited. Circ Res. 2015;116:1254‐1268. 25814686 10.1161/CIRCRESAHA.116.302317 PMC4380242

[16] Melendez GC , McLarty JL , Levick SP , et al. Interleukin 6 mediates myocardial fibrosis, concentric hypertrophy, and diastolic dysfunction in rats. Hypertension. 2010;56:225‐231. 20606113 10.1161/HYPERTENSIONAHA.109.148635 PMC2921860

[17] Si L , Xu J , Yi C , et al. Asiatic acid attenuates the progression of left ventricular hypertrophy and heart failure induced by pressure overload by inhibiting myocardial remodeling in mice. J Cardiovasc Pharmacol. 2015;66:558‐568. 26647013 10.1097/FJC.0000000000000304

[18] Nagai T , Anzai T , Kaneko H , et al. C‐reactive protein overexpression exacerbates pressure overload‐induced cardiac remodeling through enhanced inflammatory response. Hypertension. 2011;57:208‐215. 21220701 10.1161/HYPERTENSIONAHA.110.158915

[19] Hirota H , Yoshida K , Kishimoto T , Taga T . Continuous activation of gp130, a signal‐transducing receptor component for interleukin 6‐related cytokines, causes myocardial hypertrophy in mice. Proc Natl Acad Sci USA. 1995;92:4862‐4866. 7539136 10.1073/pnas.92.11.4862 PMC41807

[20] Fontes JA , Rose NR , Cihakova D . The varying faces of IL‐6: from cardiac protection to cardiac failure. Cytokine. 2015;74:62‐68. 25649043 10.1016/j.cyto.2014.12.024 PMC4677779

[21] Kaminski KA , Oledzka E , Bialobrzewska K , et al. The effects of moderate physical exercise on cardiac hypertrophy in interleukin 6 deficient mice. Adv Med Sci. 2007;52:164‐168. 18217411

[22] Lai NC , Gao MH , Tang E , et al. Pressure overload‐induced cardiac remodeling and dysfunction in the absence of interleukin 6 in mice. Lab Invest. 2012;92:1518‐1526. 22825686 10.1038/labinvest.2012.97 PMC3482286

[23] Zhao L , Cheng G , Jin R , et al. Deletion of interleukin‐6 attenuates pressure overload‐induced left ventricular hypertrophy and dysfunction. Circ Res. 2016;118:1918‐1929. 27126808 10.1161/CIRCRESAHA.116.308688 PMC4902783

[24] Li H , Xiao H , Lin L , et al. Drug design targeting protein‐protein interactions (PPIs) using multiple ligand simultaneous docking (MLSD) and drug repositioning: discovery of raloxifene and bazedoxifene as novel inhibitors of IL‐6/GP130 interface. J Med Chemy. 2014;57:632‐641. 10.1021/jm401144z 24456369

[25] Gong W , Yan M , Chen J , et al. Chronic inhibition of cyclic guanosine monophosphate‐specific phosphodiesterase 5 prevented cardiac fibrosis through inhibition of transforming growth factor beta‐induced Smad signaling. Front Med. 2014;8:445‐455. 25416030 10.1007/s11684-014-0378-3

[26] Ponikowski P , Voors AA , Anker SD , et al. 2016 ESC Guidelines for the diagnosis and treatment of acute and chronic heart failure: The Task Force for the diagnosis and treatment of acute and chronic heart failure of the European Society of Cardiology (ESC). Developed with the special contribution of the Heart Failure Association (HFA) of the ESC. Eur J Heart Fail. 2016;18: 891‐975. 27207191 10.1002/ejhf.592

[27] Barry SP , Davidson SM , Townsend PA . Molecular regulation of cardiac hypertrophy. Int J Biochem Cell Biol. 2008;40:2023‐2039. 18407781 10.1016/j.biocel.2008.02.020

[28] Heineke J , Molkentin JD . Regulation of cardiac hypertrophy by intracellular signalling pathways. Nat Rev Mol Cell Biol. 2006;7:589‐600. 16936699 10.1038/nrm1983

[29] Baker KM , Aceto JF . Angiotensin II stimulation of protein synthesis and cell growth in chick heart cells. Am J Physiol. 1990;259:H610‐H618. 2386231 10.1152/ajpheart.1990.259.2.H610

[30] Harada K , Komuro I , Shiojima I , et al. Pressure overload induces cardiac hypertrophy in angiotensin II type 1A receptor knockout mice. Circulation. 1998;97:1952‐1959. 9609089 10.1161/01.cir.97.19.1952

[31] Martino TA , Tata N , Simpson JA , et al. The primary benefits of angiotensin‐converting enzyme inhibition on cardiac remodeling occur during sleep time in murine pressure overload hypertrophy. J. Am. Coll. Cardiol. 2011;57:2020‐2028. 21565639 10.1016/j.jacc.2010.11.022

[32] Wang X , Ye Y , Gong H , et al. The effects of different angiotensin II type 1 receptor blockers on the regulation of the ACE‐AngII‐AT1 and ACE2‐Ang(1–7)‐Mas axes in pressure overload‐induced cardiac remodeling in male mice. J Mol Cell Cardiol. 2016;97:180‐190. 27210827 10.1016/j.yjmcc.2016.05.012

[33] Chen F , Chen D , Zhang Y , et al. Interleukin‐6 deficiency attenuates angiotensin II‐induced cardiac pathogenesis with increased myocyte hypertrophy. Biochem. Biophys. Res. Commun. 2017;494:534‐541. 29079193 10.1016/j.bbrc.2017.10.119

[34] Coles B , Fielding CA , Rose‐John S , et al. Classic interleukin‐6 receptor signaling and interleukin‐6 trans‐signaling differentially control angiotensin II‐dependent hypertension, cardiac signal transducer and activator of transcription‐3 activation, and vascular hypertrophy in vivo. Am J Pathol. 2007;171:315‐325. 17591976 10.2353/ajpath.2007.061078 PMC1941613

[35] deAlmeida AC , van Oort RJ , Wehrens XHT . Transverse aortic constriction in mice. J Vis Exp. 2010. 10.3791/1729 PMC3164086 20410870

[36] Furihata T , Kinugawa S , Takada S , et al. The experimental model of transition from compensated cardiac hypertrophy to failure created by transverse aortic constriction in mice. Int J Cardiol Heart Vasc. 2016;11:24‐28. 28616522 10.1016/j.ijcha.2016.03.007 PMC5441312

[37] Richards DA , Aronovitz MJ , Calamaras TD , et al. Distinct phenotypes induced by three degrees of transverse aortic constriction in mice. Sci Rep. 2019;9:5844. 30971724 10.1038/s41598-019-42209-7 PMC6458135

[38] Zeng T , Yuan J , Gan J , et al. Thrombospondin 1 is increased in the aorta and plasma of patients with acute aortic dissection. Can J Cardiol. 2019;35:42‐50. 30595182 10.1016/j.cjca.2018.11.008

[39] Lew WY , Bayna E , Molle ED , et al. Recurrent exposure to subclinical lipopolysaccharide increases mortality and induces cardiac fibrosis in mice. PLoS ONE. 2013;8:e61057. 23585870 10.1371/journal.pone.0061057 PMC3622013

[40] Liu F , Wen Y , Kang J , et al. Regulation of TLR4 expression mediates the attenuating effect of erythropoietin on inflammation and myocardial fibrosis in rat heart. Int J Mol Med. 2018;42:1436‐1444. 29845292 10.3892/ijmm.2018.3707 PMC6089778

[41] Han Y , Wang Q , Fan X , et al. Epigallocatechin gallate attenuates overloadinduced cardiac ECM remodeling via restoring T cell homeostasis. Mol Med Rep. 2017;16:3542‐3550. 28713936 10.3892/mmr.2017.7018

[42] Brown DL , Desai KK , Vakili BA , et al. Clinical and biochemical results of the metalloproteinase inhibition with subantimicrobial doses of doxycycline to prevent acute coronary syndromes (MIDAS) pilot trial. Arter Thromb Vasc Biol. 2004;24:733‐738. 10.1161/01.ATV.0000121571.78696.dc 14962945

[43] Kossakowska AE , Edwards DR , Prusinkiewicz C , et al. Interleukin‐6 regulation of matrix metalloproteinase (MMP‐2 and MMP‐9) and tissue inhibitor of metalloproteinase (TIMP‐1) expression in malignant non‐Hodgkin's lymphomas. Blood. 1999;94:2080‐2089. 10477738

[44] Kesanakurti D , Chetty C , Dinh DH , et al. Role of MMP‐2 in the regulation of IL‐6/Stat3 survival signaling via interaction with alpha5beta1 integrin in glioma. Oncogene. 2013;32:327‐340. 22349830 10.1038/onc.2012.52 PMC3368064

[45] Xie TX , Wei D , Liu M , et al. Stat3 activation regulates the expression of matrix metalloproteinase‐2 and tumor invasion and metastasis. Oncogene. 2004;23:3550‐3560. 15116091 10.1038/sj.onc.1207383

[46] Kim JY , Bae YH , Bae MK , et al. Visfatin through STAT3 activation enhances IL‐6 expression that promotes endothelial angiogenesis. Biochim Biophys Acta. 2009;1793:1759‐1767. 19751774 10.1016/j.bbamcr.2009.09.006

[47] Yang J , Liao X , Agarwal MK , et al. Unphosphorylated STAT3 accumulates in response to IL‐6 and activates transcription by binding to NFkappaB. Genes Dev. 2007;21:1396‐1408. 17510282 10.1101/gad.1553707 PMC1877751

[48] Tanida I . Autophagy basics. Microbiol Immunol. 2011;55:1‐11. 21175768 10.1111/j.1348-0421.2010.00271.x

[49] Wang X , Cui T . Autophagy modulation: a potential therapeutic approach in cardiac hypertrophy. Am J Physiol Heart Circ Physiol. 2017;313:H304‐H319. 28576834 10.1152/ajpheart.00145.2017 PMC5582915

[50] Delbridge LMD , Mellor KM , Taylor DJ , Gottlieb RA . Myocardial stress and autophagy: mechanisms and potential therapies. Nat Rev Cardiol. 2017;14:412‐425. 28361977 10.1038/nrcardio.2017.35 PMC6245608

[51] Korolchuk VI , Mansilla A , Menzies FM , Rubinsztein DC . Autophagy inhibition compromises degradation of ubiquitin‐proteasome pathway substrates. Mol Cell. 2009;33:517‐527. 19250912 10.1016/j.molcel.2009.01.021 PMC2669153

[52] Li B , Chi RF , Qin FZ , Guo XF . Distinct changes of myocyte autophagy during myocardial hypertrophy and heart failure: association with oxidative stress. Exp Physiol. 2016;101:1050‐1063. 27219474 10.1113/EP085586

[53] Zheng Q , Su H , Ranek MJ , Wang X . Autophagy and p62 in cardiac proteinopathy. Circ Res. 2011;109:296‐308. 21659648 10.1161/CIRCRESAHA.111.244707 PMC3142307

[54] Sciarretta S , Zhai P , Shao D , et al. Rheb is a critical regulator of autophagy during myocardial ischemia: pathophysiological implications in obesity and metabolic syndrome. Circulation. 2012;125:1134‐1146. 22294621 10.1161/CIRCULATIONAHA.111.078212 PMC3337789

[55] Ikeda Y , Shirakabe A , Maejima Y , et al. Endogenous Drp1 mediates mitochondrial autophagy and protects the heart against energy stress. Circ Res. 2015;116:264‐278. 25332205 10.1161/CIRCRESAHA.116.303356

[56] Gurkar AU , Chu K , Raj L , et al. Identification of ROCK1 kinase as a critical regulator of Beclin1‐mediated autophagy during metabolic stress. Nat Commun. 2013;4:2189. 23877263 10.1038/ncomms3189 PMC3740589

[57] You L , Wang Z , Li H , et al. The role of STAT3 in autophagy. Autophagy. 2015;11:729‐739. 25951043 10.1080/15548627.2015.1017192 PMC4509450

[58] Yuan X , Du J , Hua S , et al. Suppression of autophagy augments the radiosensitizing effects of STAT3 inhibition on human glioma cells. Exp Cell Res. 2015;330:267‐276. 25220423 10.1016/j.yexcr.2014.09.006

[59] Norton GR , Peterson VR , Robinson C , et al. Independent of left ventricular mass, circulating inflammatory markers rather than pressure load are associated with concentric left ventricular remodelling. Int J Cardiol. 2019;274:342‐347. 30287055 10.1016/j.ijcard.2018.09.059

[60] Xiao H , Bid HK , Chen X , et al. Repositioning Bazedoxifene as a novel IL‐6/GP130 signaling antagonist for human rhabdomyosarcoma therapy. PLoS ONE. 2017;12:e0180297. 28672024 10.1371/journal.pone.0180297 PMC5495564

[61] Chen X , Wei J , Li C , et al. Blocking interleukin‐6 signaling inhibits cell viability/proliferation, glycolysis, and colony forming activity of human medulloblastoma cells. Int J Oncol. 2018;52:571‐578. 29207075 10.3892/ijo.2017.4211 PMC5741369

[62] Ma H , Yan D , Wang Y , et al. Bazedoxifene exhibits growth suppressive activity by targeting interleukin‐6/glycoprotein 130/signal transducer and activator of transcription 3 signaling in hepatocellular carcinoma. Cancer Sci. 2019;110:950‐961. 30648776 10.1111/cas.13940 PMC6398888

