# Supplementary material for: Inhibition of Interleukin‐6/glycoprotein 130 signalling by Bazedoxifene ameliorates cardiac remodelling in pressure overload mice
Source: J Cell Mol Med. 2020 Mar 12;24(8):4748–61. doi: 10.1111/jcmm.15147 (PMC7176848; doi:10.1111/jcmm.15147)
Supplement: Supplementary file 1 — Supplementary Material [file JCMM-24-4748-s001.zip › jcmm15147-sup-0003-SupFigLegends.docx]

**Supplementary Figure 1.** LV tissues from mice after 4-week surgery were sectioned and stained with Massion’s trichrome (**A** and **B**) or picrosirius red (**C** and **D**). **A**-**D**, scale bar, 200μm, quantification of relative fibrosis area was performed by Image J. **E**-**G**, mRNA expression profiles of fibrosis-related genes in murine hearts at 4 weeks. The relative abundance of transcripts was quantified and normalized to GAPDH. (n=3 per group) Data represent means ± SEM. **P*<0.05 vs. sham group, “ns” stands for “None Significance”.

**Supplementary Figure 2.** Echocardiographic profiles of healthy mice with or without 8-week Bazedoxifene administration. **A**-**F,** LVEF, LVESID, LVESV, LVFS, LVEDID, LVEDV in two groups of healthy mice (n=5 per group). Scatterplot represents means ± SEM including individual data points. “ns” stands for “None Significance”.
