# Supplementary figures and images for: Inhibition of Interleukin‐6/glycoprotein 130 signalling by Bazedoxifene ameliorates cardiac remodelling in pressure overload mice
Source: J Cell Mol Med. 2020 Mar 12;24(8):4748–61. doi: 10.1111/jcmm.15147 (PMC7176848; doi:10.1111/jcmm.15147)

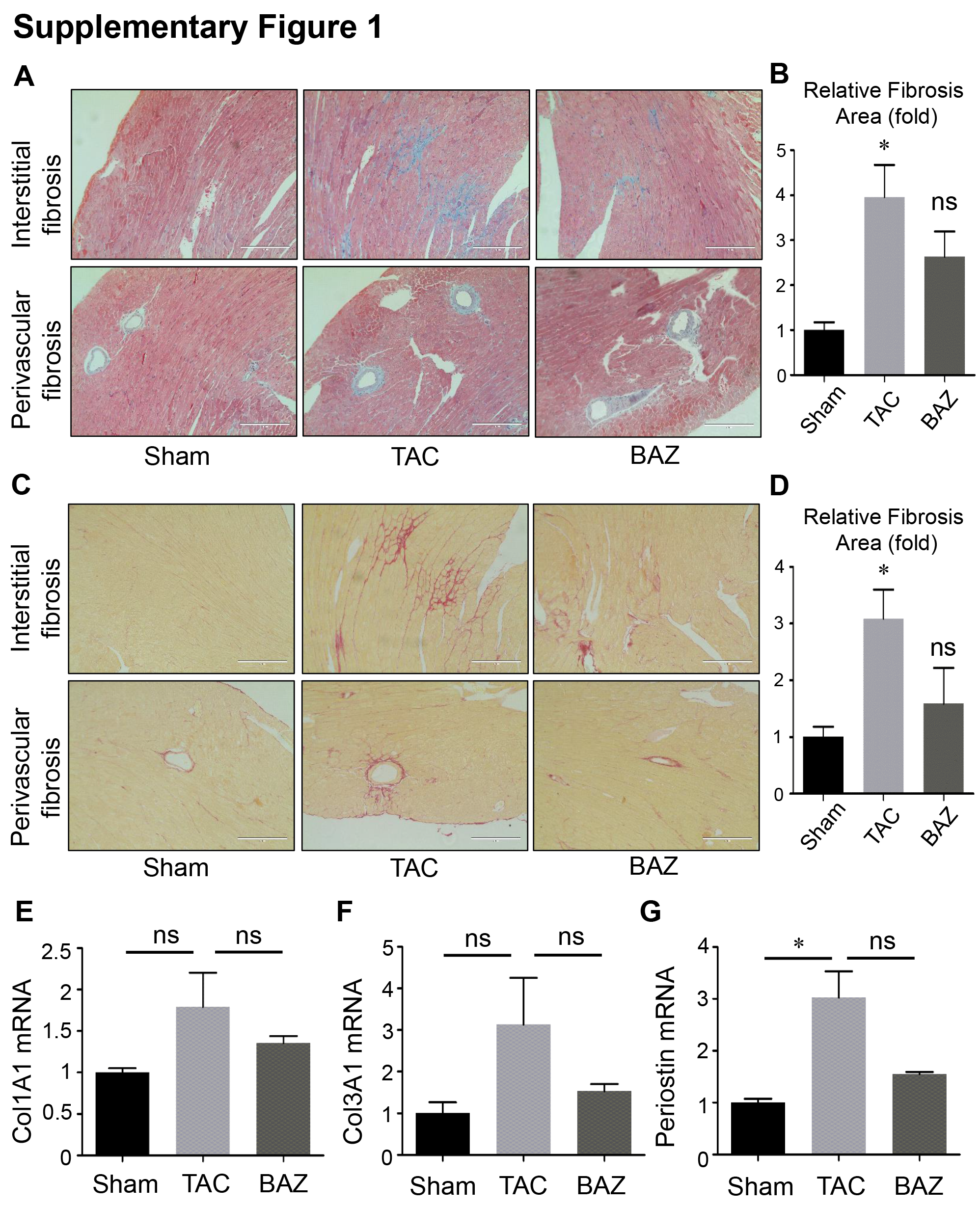

Supplement: Supplementary file 1 — Supplementary Material [file JCMM-24-4748-s001.zip › jcmm15147-sup-0001-FigS1.tif]

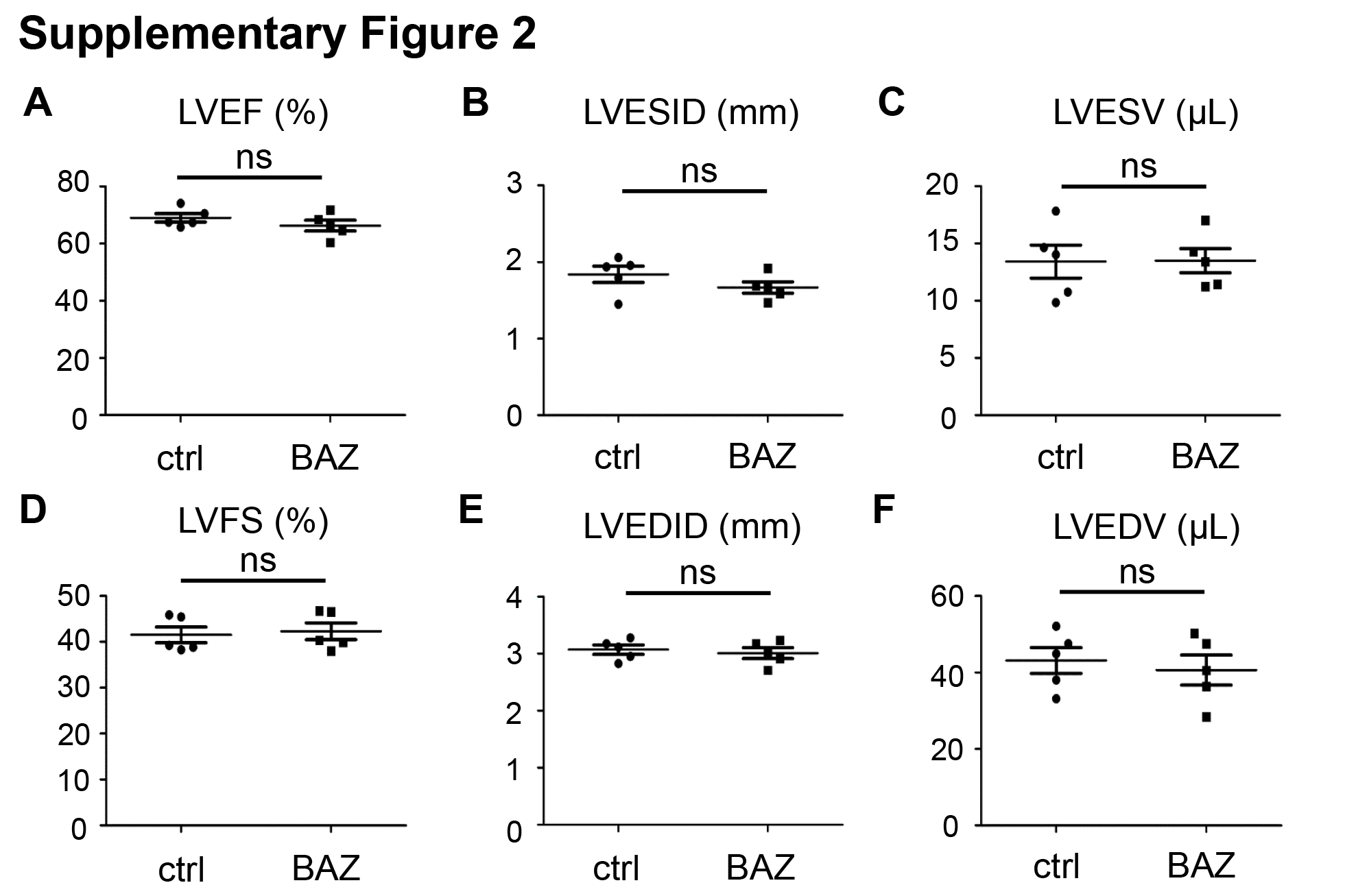

Supplement: Supplementary file 1 — Supplementary Material [file JCMM-24-4748-s001.zip › jcmm15147-sup-0002-FigS2.tif]
