# Supplementary material for: Inhibition of Interleukin‐6/glycoprotein 130 signalling by Bazedoxifene ameliorates cardiac remodelling in pressure overload mice
Source: J Cell Mol Med. 2020 Mar 12;24(8):4748–61. doi: 10.1111/jcmm.15147 (PMC7176848; doi:10.1111/jcmm.15147)
Supplement: Supplementary file 2 — Method S1 [file JCMM-24-4748-s002.docx]

Supplementary Methods

Echocardiography

Male wild-type C57BL/6J mice (8 weeks old and ≈25g) were divided into two groups: (1) healthy mice+vehicle (mice without sham nor TAC, n=5) and (2) healthy mice+BAZ (n=5). For BAZ group, mice were given a gavage of 5mg/kg BAZ (0.125mg BAZ per 100μl with 5μl DMSO respectively) while equivalent gavage without BAZ (95μl of 0.2mg/ml HPBCD and 5μl DMSO) was carried as a vehicle. After 8-week gavage the echocardiographic parameters of all mice were recorded.
